# Statistical analysis plan for hemodynamic phenotype-based, capillary refill time-targeted resuscitation in early septic shock: the ANDROMEDA-SHOCK-2 randomized clinical trial

**DOI:** 10.62675/2965-2774.20250140

**Published:** 2025-01-15

**Authors:** Nicolas Orozco, Gustavo García-Gallardo, Alexandre Biasi Cavalcanti, Tiago Mendonça dos Santos, Gustavo Ospina-Tascón, Jan Bakker, Sebastián Morales, Karla Ramos, Leyla Alegria, Jean Louis Teboul, Daniel De Backer, Antoine Vieillard-Baron, Liliana Vallecilla Fernandez, Lucas Martins de Lima, Lucas Petri Damiani, Erica Ribeiro Sady, Eliana Vieira Santucci, Glenn Hernandez, Eduardo Kattan

**Affiliations:** 1 Department of Intensive Care Medicine Fundación Valle del Lili Cali Colombia Department of Intensive Care Medicine, Fundación Valle del Lili - Cali, Colombia.; 2 HCor Research Institute HCor-Hospital do Coração São Paulo SP Brazil HCor Research Institute, HCor-Hospital do Coração - São Paulo (SP), Brazil.; 3 Department of Intensive Care Adults Erasmus MC University Medical Center Rotterdam Netherlands Department of Intensive Care Adults, Erasmus MC University Medical Center - Rotterdam, Netherlands.; 4 Departamento de Medicina Intensiva Facultad de Medicina Pontificia Universidad Católica de Chile Santiago Chile Departamento de Medicina Intensiva, Facultad de Medicina, Pontificia Universidad Católica de Chile - Santiago, Chile.; 5 Faculté de Médecine Paris-Saclay University Le Kremlin-Bicêtre France Faculté de Médecine, Paris-Saclay University - Le Kremlin-Bicêtre, France.; 6 Department of Intensive Care CHIREC Hospitals Université Libre de Bruxelles Brussels Belgium Department of Intensive Care, CHIREC Hospitals, Université Libre de Bruxelles - Brussels, Belgium.; 7 Medical and Surgical ICU University Hospital Ambroise-Paré Boulogne-Billancourt France Medical and Surgical ICU, University Hospital Ambroise-Paré - Boulogne-Billancourt, France.; 8 Centro de Investigaciones Clínicas Fundación Valle del Lili Cali Colombia Centro de Investigaciones Clínicas, Fundación Valle del Lili -Cali, Colombia.

**Keywords:** Septic shock, Phenotype, Critical care, Perfusion

## Abstract

**Background:**

ANDROMEDA-SHOCK 2 is an international, multicenter, randomized controlled trial comparing hemodynamic phenotype-based, capillary refill time-targeted resuscitation in early septic shock to standard care resuscitation to test the hypothesis that the former is associated with lower morbidity and mortality in terms of hierarchal analysis of outcomes.

**Objective:**

To report the statistical plan for the ANDROMEDA--SHOCK 2 randomized clinical trial.

**Methods:**

We briefly describe the trial design, patients, methods of randomization, interventions, outcomes, and sample size. We portray our planned statistical analysis for the hierarchical primary outcome using the stratified win ratio method, as well as the planned analysis for the secondary and tertiary outcomes. We also describe the subgroup and sensitivity analyses. Finally, we provide details for presenting our results, including mock tables, baseline characteristics, and the effects of treatments on outcomes.

**Conclusion:**

According to best trial practices, we report our statistical analysis plan and data management plan prior to locking the database and initiating the analyses. We anticipate that this practice will prevent analysis bias and improve the utility of the study’s reported results.

## INTRODUCTION

Septic shock is a prevalent and lethal disease, with estimated mortality rates of approximately 40%.^1^Uncontrolled host response to infection results in a series of progressive derangements in circulatory homeostasis, including macro- and microcirculatory derangements, which lead to progressive tissue hypoperfusion, multiorgan dysfunction, and death.^2^

Prompt hemodynamic resuscitation is key for deactivating this vicious cycle. Tailored interventions such as fluids, vasopressors, and inotropes may improve this consumption‒delivery imbalance and possibly reverse this process.^3^However, each intervention has a narrow therapeutic index and is not free of adverse effects. Conversely, recently, the wide heterogeneity of hemodynamic patterns (i.e., vasoplegia, hypovolemia or cardiac dysfunction) and the emergence of peripheral perfusion as a relevant target of resuscitation have increased our understanding of the kinetics of septic shock resuscitation.^4,5^ Thus, personalization of therapy according to key macro- and microcirculatory parameters could aid in further improving patient-centered outcomes rseptic shock resuscitation.

The ANDROMEDA-SHOCK-2 is an investigator-led multicenter randomized controlled trial that integrates these physiological precepts into a resuscitation algorithm for early septic shock.^6^The objective of this manuscript is to outline the statistical analysis plan (SAP) of the trial with the aim of preventing sources of bias from exploratory analyses after the results are available. The SAP was developed following recommended guidelines prior to locking the trial database and starting analyses.

Our primary objective is to determine whether capillary refill time (CRT)-targeted resuscitation based on clinical hemodynamic phenotyping is associated with a decrease in a hierarchical composite outcome within 28 days after randomization, which includes mortality, time to cessation of vital support, and length of hospital stay, compared with standard care in patients with early septic shock.

Our secondary objectives are to determine whether CRT-targeted resuscitation based on clinical hemodynamic phenotyping is associated with a decrease in all-cause mortality, more organ support-free days, and a decreased length of hospital stay within 28 days after randomization compared with standard care in patients with early septic shock.

## METHODS

### Trial design

**Figure 1:**
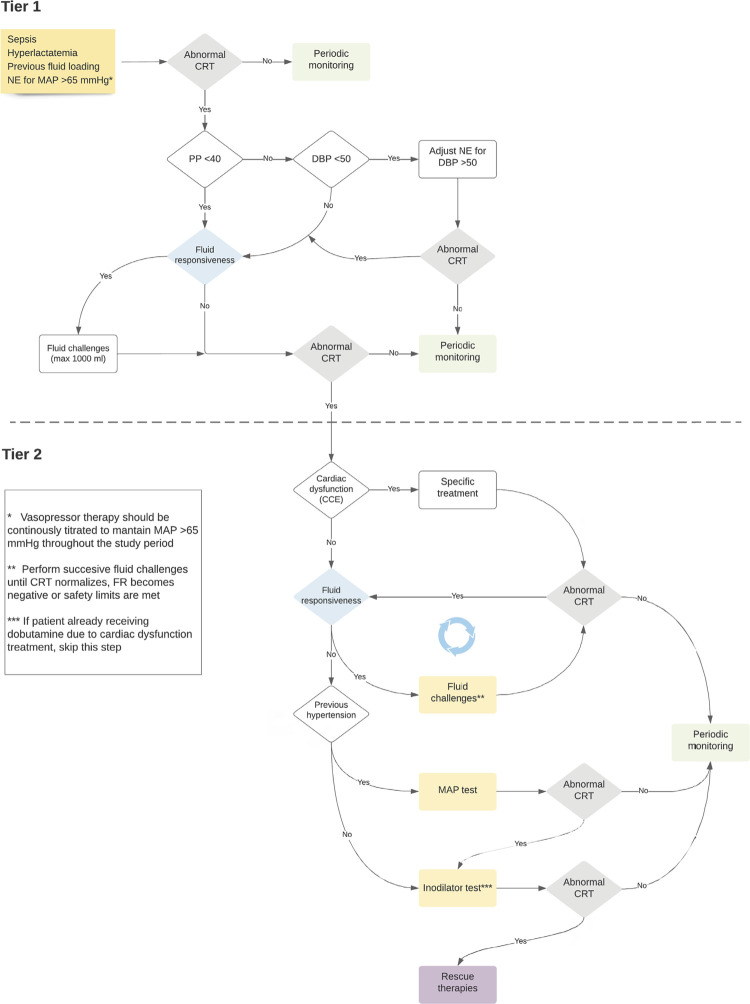
Resuscitation strategies of the Intervention Group.

ANDROMEDA-SHOCK-2 is a prospective, multicenter, parallel-group, randomized trial that compares a 6-hour protocol of hemodynamic phenotype-based, CRT-targeted resuscitation *versus* standard care resuscitation in patients with septic shock. The trial protocol (version 1.0 from September 2021) was published and is registered with ClinicalTrials.gov (NCT05057611). The study is being conducted in 81 intensive care units (ICUs) in Latin America, North America, Europe, and Asia. The study was approved by the Ethics Committees of all the participating institutions. The main study interventions are summarized in figure 1.

NE - norepinephrine; MAP - mean arterial pressure; CRT - capillary refill time; PP - pulse pressure; DBP - diastolic blood pressure; FR - fluid responsiveness; CCE - critical care echocardiography.

### Randomization

Eligible patients are randomly allocated to the intervention (CRT-P) or control (standard care) groups. A randomization sequence with an allocation of 1:1 is generated by a computer program. Study group assignment is achieved by means of randomized permuted blocks of variable size with stratification by center. Allocation concealment is maintained by means of central randomization via the web via Castor EDC®.

Because this is a nonpharmacological intervention, blinding the medical team is not feasible.

### Study interventions

Study interventions encompass a description of the general management for both groups, the CRT-P study protocol, the standard care group, and safety measures, all of which were previously delineated and elaborated upon in the literature.^6^

### Sample size

Given that the intervention arm includes several new steps aimed at better tailoring fluid administration, we expect a further decrease in the use of fluids. Based on ANDROMEDA-SHOCK data, this new algorithm could reduce fluid administration in at least 60% of these patients. Previous studies have shown that a reduction in fluid administration is associated with better outcomes. Thus, we estimate a 6% absolute reduction in mortality. The estimates of the number of days needing life support truncated at 28 days and the length of hospital stay in the Control Group were based on data from the ANDROMEDA-SHOCK Control Group.

For the Control Group, we assumed a 28-day mortality of 39%, number of days needing life support truncated in 28 days among survivors of 5.6 (standard deviation [SD], 6.7) and a length-of-hospital stay truncated in 28 days among survivors of 15.6 days (SD, 10.1). We considered that the experimental group treatment would reduce mortality to 33%, shorten the number of days of life support use among survivors to 4.3 (SD, 6.2) and shorten the length of hospital stay among survivors to 14 days (SD, 9.9).The length of hospital stay and the number of days needing life support were simulated assuming a beta-binomial distribution within 28 days via parameters acquired in the ANDROMEDA-SHOCK trial.^7^

Using the win ratio method with the three components listed above, with 1,500 patients enrolled, the trial will have a power of 88% to show superiority in the win ratio outcome for a two-sided α of 0.05. The study power was estimated based on 1,000 replications.

### Framework

The study design aims to demonstrate the superiority of the CRT-targeted strategy over the standard care strategy in terms of the hierarchical primary outcome (all-cause mortality within 28 days, time to cessation of vital support within 28 days, and length of hospital stay truncated at Day 28) using the win ratio method.

### Statistical interim analyses

Interim analyses are conducted after the inclusion of the first 200 patients and at 75% of the sample size (1,125 patients). Only the independent Data Safety Monitoring Board (DSMB) has access to the results of those analyses. The DSMB is formed by independent epidemiologists, intensivists, and a senior statistician. The DSMB established guidelines for stopping the trial for safety or efficacy. Stopping for safety will be considered if a p value < 0.01 comparing the primary outcome reveals harm for the experimental group compared with the Control Group in any of the interim analyses. Conversely, stopping for efficacy will be considered in the analysis with 75% of the sample size if there is a p value < 0.001 in favor of the experimental *versus* control treatment. According to the Haybittle–Peto rule, considering the strict p value threshold specified for the interim analysis (< 0.001), no further penalization is required for the final study analysis alpha value.

### Timing of final analysis

All outcomes will be analyzed simultaneously after we complete the 90-day follow-up of all patients, and the database will be locked.

### Timing of outcome assessment

We will assess outcomes at 6, 24, 48, and 72 hours; during the first 7 days until ICU discharge; at 28 and 90 days; and at ICU and hospital discharge.

### Statistical analysis principles

We will report two-sided p values for the primary outcome main analysis. If the experimental group strategy is superior to the Control Group for the primary outcome (p value < 0.05), we will test the treatment effect on the secondary outcomes in a hierarchical fashion at a significance level of 0.05. Ninety-five percent confidence intervals will be presented for all effect estimates. P values will not be presented for tertiary outcomes or other analyses. Owing to the risk of Type I error from numerous comparisons, the results for tertiary outcomes and other analyses should be considered exploratory.

### Adherence and protocol deviations

Protocol adherence will be described in a table reporting the numbers and percentages of nonadherence to the Intervention Group protocol and good clinical practice principles.

Protocol deviations will be assessed by a central clinical monitor for all centers. Major deviations are defined as incorrect inclusion, failure of informed consent, or inadequate resuscitation procedures regarding the CRT-P flowchart during the study period (first 6 hours). Minor deviations are defined as changes in the resuscitation procedures but with a clinically valid argument. This is shown in table 1S and figure 1S (Supplementary Material).

### Analysis populations

Analysis of the treatment effect on the primary outcome will consider an intention-to-treat population, defined as all participants who fit the inclusion and exclusion criteria, are randomized, and for whom there is consent for the use of data.^8-10^We do not anticipate substantial loss of follow-up for the primary outcome data. Nevertheless, eventual missing outcome data will be imputed via the chained equations method considering site, age, and baseline Acute Physiology and Chronic Health Evaluation (APACHE) II score. The conclusion of the trial will be based on the intention-to-treat analysis.

Analysis of secondary and other outcomes will consider the same intention-to-treat population, although no imputation will be carried out for patients lost to follow-up.

### Analysis methods

The distribution of continuous variables will be assessed via visual inspection of histograms, Q‒Q plots, and normality tests such as D’Agostino‒Pearson tests. Variables with a normal distribution will be shown as the means and SDs, and those without a Gaussian distribution will be presented as medians and interquartile ranges (IQRs). For categorical variables, counts and percentages will be calculated whenever appropriate. This is shown in tables 1 and 2 (dummy tables), and tables 2S to 8S (Supplementary Material).

### Statistical software

Analysis will be performed via R version 4.2.3 software.

### Participant flow

#### Screening data

An active daily screening for potentially eligible patients is performed across all participating ICUs. Patients meeting the septic shock criteria upon admission or meeting these criteria during their ICU stay, as defined by the Sepsis-3 consensus will undergo screening.^11^Inclusion or exclusion decisions are communicated to the study coordinating center (SCC) through a designated internet-based tool (form), with reasons for exclusion documented. Centers are required to report included or excluded subjects to the SCC on a weekly basis.

#### Eligibility

Consecutive adult patients (≥ 18 years old) with septic shock admitted to the intensive care unit will be considered eligible. Septic shock is defined as suspected or confirmed infection, hyperlactatemia (≥ 2.0mmol/L) and the need for vasopressors due to refractory hypotension.^6^

The exclusion criteria include a duration of more than 4 hours after the onset of septic shock, anticipated surgery or dialysis procedure during the first 6 hours after septic shock diagnosis, active bleeding, do-not-resuscitate status, child B-C cirrhosis, underlying disease process with a life expectancy < 90 days and/or the attending clinician deems aggressive resuscitation unsuitable, refractory shock (defined as high risk of death within 24 hours), pregnancy, concomitant severe respiratory distress syndrome, and the inability to accurately assess CRT.

Patients deemed eligible may not be randomized in the case of consent denial, logistical reasons, lack of approval to participate from the attending physician (MD), or a delay in the transfer process leading to a loss of the 4-hour inclusion window.

#### Recruitment

**Figure 2:**
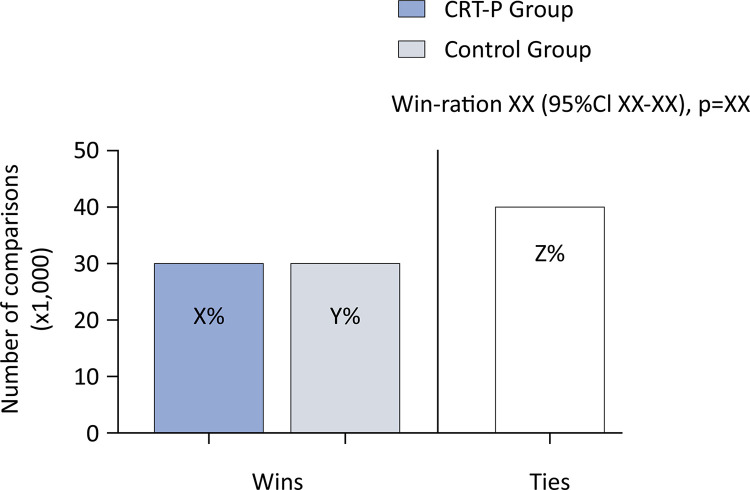
CONSORT flow.

Details for the CONSORT flow diagram are presented in figure 2.

ARDS - acute respiratory distress syndrome; CRT - capillary refill time; MD - physician.

#### Withdrawal/follow-up

We will document the number of patients whose consent for trial participation is withdrawn either by the patient or his or her legal representative. When consent is withdrawn for trial participation, we nevertheless attempt to obtain consent for the collection and analysis of follow-up data. These cases will also be reported.

#### Baseline patient characteristics

The baseline characteristics recorded during the trial will be presented in table 1 (dummy).

**Table 1:** Patient characteristics at randomization

Characteristic	CRT-P guided resuscitation (n = xxx)	Usual care (n = xxx)
Demographics		
Age (years)	xx (xx – xx)	xx (xx – xx)
Sex		
Men	xx (xx)	xx (xx)
Women	xx (xx)	xx (xx)
Body mass index	xx (xx – xx)	xx (xx – xx)
APACHE II	xx (xx – xx)	xx (xx – xx)
Baseline SOFA score	xx (xx – xx)	xx (xx – xx)
Charlson Comorbidity Index	xx (xx – xx)	xx (xx – xx)
Time from hypotension to antibiotic start (hours)	xx (xx – xx)	xx (xx – xx)
Time from meeting entry criteria to randomization (hours)	xx (xx – xx)	xx (xx – xx)
Comorbidities		
Hypertension	xx (xx)	xx (xx)
Diabetes	xx (xx)	xx (xx)
Chronic obstructive pulmonary disease	xx (xx)	xx (xx)
Chronic renal disease	xx (xx)	xx (xx)
Hematological cancer	xx (xx)	xx (xx)
Nonhematological Cancer	xx (xx)	xx (xx)
Immunosuppression	xx (xx)	xx (xx)
Liver disease	xx (xx)	xx (xx)
Heart failure	xx (xx)	xx (xx)
Coronary artery disease	xx (xx)	xx (xx)
Source of infection		
Abdominal	xx (xx)	xx (xx)
Lung	xx (xx)	xx (xx)
Urinary tract	xx (xx)	xx (xx)
Soft tissues	xx (xx)	xx (xx)
Primary bacteremia	xx (xx)	xx (xx)
Other	xx (xx)	xx (xx)
Unknown	xx (xx)	xx (xx)
Characteristics at randomization		
Resuscitation fluids at randomization (mL)	xx (xx – xx)	xx (xx – xx)
Supplemental oxygen	xx (xx)	xx (xx)
Mechanical ventilation		
Invasive	xx (xx – xx)	xx (xx – xx)
Noninvasive	xx (xx – xx)	xx (xx – xx)
Lactate > 4.0 mmol/L	xx (xx)	xx (xx)
Lactate level (mmol/L)	xx (xx – xx)	xx (xx – xx)
Mottling score		
Capillary refill time (seconds)	xx (xx – xx)	xx (xx – xx)
Norepinephrine dose* (mcg/kg/minute)	x.xx (x.xx - x.xx)	x.xx (x.xx - x.xx)
Vasopressin use	xx (xx)	xx (xx)
Venous-to-arterial carbon dioxide difference (mmHg)	xx (xx – xx)	xx (xx – xx)
Central venous oxygen saturation	xx (xx – xx)	xx (xx – xx)

CRT-P - Intervention Group; APACHE - Acute Physiology and Chronic Health Evaluation; SOFA - Sequential Organ Failure Assessment. * Expressed as a norepinephrine base.

## Analysis

### Outcome definitions

#### Primary outcome

The primary outcome of the study is a hierarchical composite outcome: (1) all-cause mortality within 28 days, (2) time to cessation of vital support truncated on Day 28, and (3) length of hospital stay truncated on Day 28.

#### Secondary outcomes

The following are the secondary outcomes assessed in a hierarchical fashion: 1) all-cause mortality within 28 days; 2) vital support-free days within 28 days, defined as the number of days between randomization and 28 days after randomization in which the patient is alive and does not require cardiovascular, respiratory, and/or renal support (a value of zero will be assigned to patients who die within 28 days, regardless of their life support status) and 3) length of hospital stay, truncated on Day 28.

#### Tertiary outcomes

The tertiary clinical outcomes are as follows: all-cause mortality within 90 days after randomization, length of hospital stay, length of ICU stay, time to cessation of vasopressor support, time to cessation of mechanical ventilation (MV), time to cessation of renal replacement therapy (RRT), vasopressor support-free days, MV-free days, RRT-free days, variation in Sequential Organ Failure Assessment (SOFA) score, variation in creatinine-based Kidney Disease: Improving Global Outcomes (KDIGO) stage, volume of resuscitation fluids, net fluid balance, evolution of CRT, evolution of lactate levels, evolution of central venous pressure (CVP), evolution of central venous oxygen saturation (ScvO2), and evolution of the central venous to arterial carbon dioxide difference (delta pCO2(v-a)).

## Primary outcome analysis

This outcome will be analyzed via the stratified win ratio method, with treatment used as a fixed effect and the APACHE II score used as a stratifying variable.^12,13^All the patients in the CRT-P group will be compared with all the patients in the Control Group following the hierarchical primary outcome. Thus, in each pair, the comparison will focus first on mortality (1). If only one patient dies, the surviving patients will sum a win. If both patients in a pair die, it will be deemed a tie, leading to no further comparisons for this pair. If both patients survive past 28 days, the analysis will shift to the time to cessation of vital support (2). A win will be assigned to the group containing the patient with the shortest time to cessation of vital support. If the duration (in days) is equal within the pair, then the length of hospital stay (3) will be compared. A win will be counted for the treatment group with the patients having the shortest hospital stay. If the length is identical, it will be marked as a tie.

The win ratio is calculated as:


$$\text{ Win ratio }\frac{\text{ Number of wins in the experimental group }}{\text{ Number of wins in the Control Group }}$$

**Figure 3:**
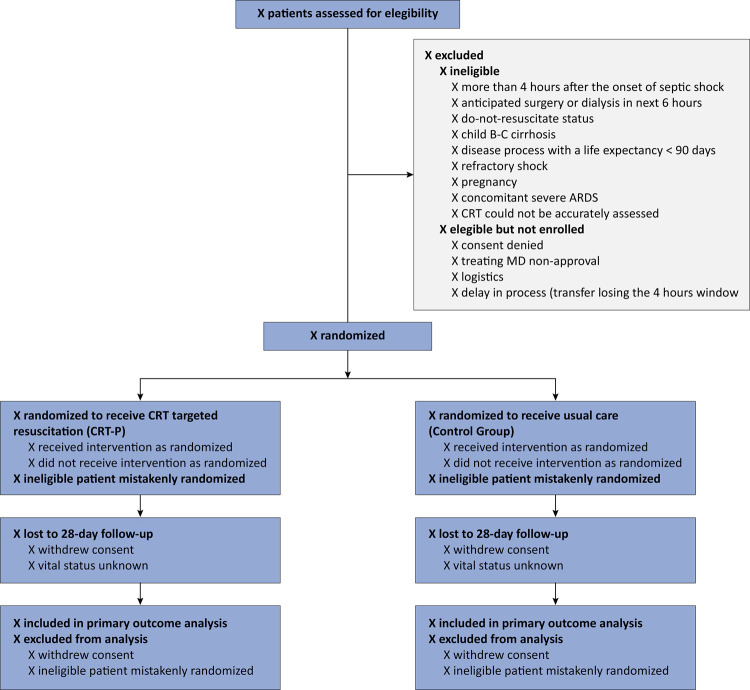
Primary outcome (win ratio) analysis dummy figure.

The bootstrap resampling method will be used to calculate the 95% confidence interval (95%CI) for the win ratio and p value for the hypothesis test and is reported in figure 3 (dummy). The results will be reported as win ratios with 95%CIs and p values. The win ratio is interpreted as follows: in any pair of patients, one treated with the experimental intervention and the other with the control intervention, and as long as there is no tie, the win ratio is the probability that the treated patient will have a better outcome.

CRT-P - Intervention Group.

Compared with the original analysis approach described by Pocock et al.,^12^our analysis has modifications. In the original analysis, death was assessed as a time-to-event variable, and even if both patients of a pair died, the one who lived longer would win. In our study, we consider death within 28 days as a binary event, and if both patients in a pair die, we consider this a tie. This is based on the assumption that a later death within a 28-day period would not be considered a benefit from patients’ perspectives.

## Secondary outcome analysis

The effect on 28-day all-cause mortality will be assessed with a Cox proportional hazard model adjusted for baseline APACHE II score and presented as hazard ratios with 95%CI, similar to the analysis implemented in the ANDROMEDA SHOCK trial.^14^The effect on vital support-free days within 28 days will be assessed with a cumulative logistic model adjusted for the baseline APACHE II score and presented as odds ratios with 95%CIs. The length of hospital stay, which is truncated on Day 28, will be estimated with generalized linear models adjusted for the APACHE II score using the distribution that best fits the data. The results will be presented in table 2 (dummy). The results of residual and QQ-plot analysis will be published in the supplementary manuscript to increase transparency.

**Table 2:** Outcomes

Characteristic	CRT-P guided resuscitation (n = xxx)	Usual care (n = xxx)	Effect estimate	Effect size (95%CI)	p value
Primary outcome					
Hierarchical composite outcome at 28 days*	xxx / xxx (xx.x)	xxx / xxx (xx.x)	Win ratio	x.xx (x.xx to x.xx)	.xx
Secondary outcomes					
All-cause mortality within 28 days	xxx / xxx (xx.x)	xxx / xxx (xx.x)	Hazard ratio	x.xx (x.xx to x.xx)	.xx
Vital support-free days within 28 days	xx.x (xx.x; xx.x)	xx.x (xx.x; xx.x)	TBD†	x.xx (x.xx to x.xx)	.xx
Length of hospital stay (truncated at 28 days)	xx.x (xx.x; xx.x)	xx.x (xx.x; xx.x)	TBD†	x.xx (x.xx to x.xx)	.xx

CRT-P - Intervention Group; 95%CI - 95% confidence interval; * Composite Hierarchical Outcome: all-cause mortality, then time to cessation of vital support, and length of hospital stay, truncated at 28 days. † A generalized linear model with the distribution that best fits the data will be adjusted; thus, the effect estimate measure will be subsequently defined.

## Tertiary outcome analysis

All-cause mortality within 90 days will be assessed with a Cox proportional hazard model adjusted for the baseline APACHE II score. The effects on the length of hospital stay, length of ICU stay, time to cessation of vasopressor support, time to cessation of MV, and time to cessation of RRT will be estimated with generalized linear models using the distribution that best fits the data and adjusted for the baseline APACHE II score. Vasopressor support-free days, MV-free days, and RRT-free days will be assessed with a cumulative logistic model adjusted for the baseline APACHE II score.^15,16^Effects on repeated measures of organ dysfunction at 7 days (SOFA), CRT and lactate levels will be analyzed via mixed models with patients as random effects, treatment groups as fixed effects, and treatment‒time interactions while adjusting for the baseline SOFA score. The effect will be presented as the difference between the slopes of the groups or, equivalently, the treatment‒time interaction. The effects on other continuous outcomes, such as length of hospital stay and length of ICU stay, will be calculated with generalized linear models with the distribution that best fits the data. Adjustments for baseline APACHE II score, evolution volume resuscitation fluid, and net fluid balance will also be assessed with generalized linear models with the distribution that best fits the data and no adjustments for baseline covariates. For all continuous outcomes for which we will define the best distribution at the time of statistical analysis, the criteria will involve assessing which distribution best fits the data via residual and QQ-plot analysis, which will be published in the supplementary manuscript to increase transparency.

## Heterogeneity of treatment effects

We will assess whether treatment varies according to baseline patient characteristics via conventional subgroup analysis and risk modeling.^17^

The effect of the treatment on the primary hierarchical composite outcome will be assessed according to the following subgroups: age (< or ≥ 65 years), APACHE II score (< or ≥ 25 points), SOFA score (< or ≥ 10 points), baseline lactate level (< or ≥ 4mmol/L), baseline CRT (< or ≥ 3 seconds), origin of septic shock (pulmonary, abdominal, urinary, soft tissue *versus* bacteraemia *versus* other), and invasive mechanical ventilation at inclusion.

We will assess the heterogeneity of treatment effects by risk modeling through analysis of the win ratio according to quintiles of APACHE-2 scores.^17^

## Harms

The primary, secondary and tertiary outcomes intended to reflect potential harms resulting from using the CRT-P *versus* the standard resuscitation approach for managing septic shock are shown in tables 2 to 4 (Supplementary Material).

## Preplanned *post hoc* analysis

Considering the granularity of the data registered, multiple preplanned post hoc analyses will be performed from the ANDROMEDA-SHOCK-2 trial database. Our assessments include the following: an assessment of the evolution of fluid responsiveness over time and according to cumulative fluids administered; an assessment of the prevalence of use, achievement and impact of the three hemodynamic interventions (DAP test, MAP test, and inodilator test) on tissue perfusion; a Bayesian reanalysis of the primary outcome; a per-protocol analysis; an analysis of the kinetics of CRT evolution during the resuscitation timeframe; an assessment of the need for echocardiography; an assessment of the prevalence and patterns of cardiac dysfunction; a VExUS assessment after the study period and its relationship with acute kidney injury; and an assessment of the interactions between study groups, comparing high *versus* low recruiters and the first 25% *versus* the last 25% of patients recruited. We plan to carry out effect-modeling analysis via machine learning models to assess the effects of the experimental *versus* control strategies on 28-day mortality.^18^

## CONCLUSION

In accordance with best practices, we report our statistical analysis plan and data management plan prior to locking ANDROMEDA-SHOCK-2 study’s database. We anticipate that this practice will prevent analysis bias and improve the utility of the study’s reported results.
